# COVID-19 Vaccination in Patients With Cancer and Patients Receiving HSCT or CAR-T Therapy: Immune Response, Real-World Effectiveness, and Implications for the Future

**DOI:** 10.1093/infdis/jiad174

**Published:** 2023-08-04

**Authors:** Victoria G Hall, Benjamin W Teh

**Affiliations:** Sir Peter MacCallum Department of Oncology, University of Melbourne, Parkville, Victoria, Australia; Department of Infectious Diseases, Peter MacCallum Cancer Centre, Melbourne, Victoria, Australia; Sir Peter MacCallum Department of Oncology, University of Melbourne, Parkville, Victoria, Australia; Department of Infectious Diseases, Peter MacCallum Cancer Centre, Melbourne, Victoria, Australia

**Keywords:** chimeric-antigen receptor T-cell therapy, hematologic malignancies, hematopoietic stem cell transplant, mRNA COVID-19 vaccines, solid tumors

## Abstract

Patients with cancer demonstrate an increased vulnerability for infection and severe disease by SARS-CoV-2, the causative agent of COVID-19. Risk factors for severe COVID-19 include comorbidities, uncontrolled disease, and current line of treatment. Although COVID-19 vaccines have afforded some level of protection against infection and severe disease among patients with solid tumors and hematologic malignancies, decreased immunogenicity and real-world effectiveness have been observed among this population compared with healthy individuals. Characterizing and understanding the immune response to increasing doses or differing schedules of COVID-19 vaccines among patients with cancer is important to inform clinical and public health practices. In this article, we review SARS-CoV-2 susceptibility and immune responses to COVID-19 vaccination in patients with solid tumors, hematologic malignancies, and those receiving hematopoietic stem cell transplant or chimeric-antigen receptor T-cell therapy.

Since the emergence of SARS-CoV-2, the causative agent of COVID-19, the literature has demonstrated an increased vulnerability for infection and severe disease among patients with cancer [1, 2]. In patients with solid tumors, immune dysregulation may arise due to vascular endothelial growth factor overexpression inhibiting immune cell development and function [3]. From a large prospective cohort of patients with solid tumors, researchers found that age and sex rather than receipt of active chemotherapy were risk factors for severe COVID-19, similar to the general population [4]. In patients with hematologic malignancies, susceptibility for infection may relate to immunosuppression from their underlying disease and/or receipt of anticancer therapy [2, 5–7]. Other risk factors for COVID-19 infection or severe disease include comorbidities, status of disease (controlled vs uncontrolled), and current line of treatment ([neo]adjuvant, first line, or second line and beyond) [8–10]. In addition, recipients of hematopoietic stem cell transplant (HSCT) or chimeric-antigen receptor T-cell therapy (CAR-T) are at high risk for SARS-CoV-2 infection and severe COVID-19 disease due to dysregulation of immune cell populations via broad myelo- and lymphotoxicity from preparatory conditioning regimens and slow recovery of immunity and the impact of prior treatment regimens [6, 11, 12]. A higher immunodeficiency score index group, older age, and poor performance status have also been associated with a higher risk of severe COVID-19 in patients following HSCT [13, 14].

The emergence of COVID-19 vaccines has afforded some level of protection against infection and severe disease among patients with cancer; however, decreased immunogenicity and real-world effectiveness was observed among patients with solid tumors and hematologic malignancies [6, 11, 15–17]. Thus, understanding and characterizing the immune response to increasing doses or differing schedules of COVID-19 vaccines among patients with cancer are important to better inform clinical and public health practices in this population moving forward. In this study, we review SARS-CoV-2 susceptibility and immune responses to COVID-19 vaccination in patients with solid tumors, hematologic malignancies, and those receiving HSCT or CAR-T.

## EPIDEMIOLOGY AND BURDEN OF COVID-19 AMONG PATIENTS WITH CANCER AND THE IMPACT OF VACCINATION

Analyses from the National Center for Advancing Translational Sciences National COVID Cohort Collaborative identified 398 579 adult patients with cancer in the United States, 63 413 (15.9%) of whom had a COVID-19 diagnosis between January 1, 2020 and March 25, 2021 (vaccination status was not indicated but due to timing of the analysis, unvaccinated status can likely be assumed) [12]. COVID-19 was most prevalent in patients with skin (14.9%), breast (14.2%), prostate (12.3%), hematologic (12.3%), and gastrointestinal cancers (8.8.%) [12]. Between December 2019 and June 2020, the estimated global prevalence of COVID-19 among patients with cancer was 7% but ranged from 4% to 22% depending on the country and/or region [5].

Among patients with cancer, a current diagnosis of COVID-19 is associated with increased risk of mortality. A cohort study characterized COVID-19 outcomes in patients with active or previous malignancy from the COVID-19 and Cancer Consortium (CCC19) database between March 17 and April 16, 2020, and identified 928 patients, 26% of whom had severe COVID-19 illness and 13% died [7]. In addition, before the availability of COVID-19 vaccines, a study in the United Kingdom reported a mortality rate of 30.6% in patients with cancer and COVID-19, citing COVID-19 as the cause of death in 92.5% of these cases [18]. Patients with hematologic malignancies, and those receiving autologous or allogeneic HSCT or CAR-T, are at higher risk for severe COVID-19 and COVID-19–related mortality relative to other cancer populations [6, 12, 14, 19]. For instance, in patients with hematologic malignancies and those receiving CAR-T, COVID-19–related mortality rates of up to 34% and 41% have been reported (before the availability of COVID-19 vaccines or when vaccination status was not reported, respectively), which is up to 40 times greater than the COVID-19 mortality rate for the general population, depending on the country and type of underlying hematologic malignancy [17, 20]. Overall, findings suggest that the risks of SARS-CoV-2 infection and severe COVID-19 is dependent on the type of cancer, with higher risk for patients with hematologic malignancies [20–24].

Breakthrough infections and severe outcomes are more likely to occur in COVID-19–vaccinated patients with cancer than in vaccinated individuals without cancer, with the highest risk in patients with hematologic malignancies and those receiving systemic antineoplastic agents (specifically during the delta and omicron wave) [23, 24]. Widespread uptake of COVID-19 vaccines has substantially reduced the mortality rate among patients with cancer [25, 26]. Overall, these results highlight the poor outcomes among patients with cancer infected with COVID-19, especially in patients with hematologic malignancies and those receiving CAR-T, and emphasize the importance of vaccination in this population.

## SARS-CoV-2 VACCINE IMMUNOGENICITY STUDIES IN PATIENTS WITH CANCER

### Humoral Immune Responses

Many studies have focused on SARS-CoV-2 antibody response to the receptor binding domain (RBD) or spike protein and have demonstrated that patients with cancer have lower COVID-19 vaccine seropositivity rates than the general adult population [27–29]. Although patients with solid tumors typically have higher seropositivity rates compared with patients with hematologic malignancies, or those undergoing HSCT or CAR-T, antibody levels are still lower than in individuals without cancer [30–33]. Furthermore, receipt of active cancer therapy can impact immune responses after COVID-19 vaccination (Table 1 [30, 34, 35]). In particular, B cell-depleting therapies (including cluster of differentiation [CD]-20 monoclonal antibodies) and the use of Bruton's tyrosine kinase inhibitors and Janus kinase inhibitors have been associated with reduced seropositivity rates [24, 30, 34–37]. In contrast, endocrine therapy, tyrosine kinase inhibitors for solid tumors, proteasome inhibitors, and immunomodulatory drugs have not demonstrated an impact on seroresponse [30, 34, 35].

**Table 1.** Level of Response to COVID-19 Vaccination [30, 34, 35]

Cancer Treatment	Impact of Treatment on RBD-Antibody Response^a^	Additional Considerations
Endocrine therapy	Low	**…**
Tyrosine kinase inhibitor	Low	TKIs used for the treatment of solid tumors do not appear to impair humoral responses to COVID-19 vaccination
Proteasome inhibitors	Low	…
Immunomodulatory drugs	Low	…
Poly(ADP-ribose) polymerase inhibitors	Low	…
Cyclin-dependent kinase 4 and 6 (CDK4/6) inhibitors	Low	…
Immune checkpoint inhibitors	Low-moderate	Most patients were able to mount an adequate antibody response after 2 doses of vaccines while receiving ICIs
Conventional chemotherapy	Low-moderate	Recent chemotherapy (28 days to within 6 months of vaccination) has been identified as a risk factor for lower serologic response
Corticosteroids	Low-moderate	Impact determined by corticosteroid dose; high-dose steroid use is a risk factor for reduced serologic response
Hematopoietic stem cell transplant	Moderate	Serologic response is dependent on timing of vaccination relative to transplant and use of any concurrent immunosuppressive treatments
Janus kinase inhibitor	Moderate	Studies suggest lower antibody levels with ruxolitinib compared to non-JAKi-treated patients with MPN
Cluster of differentiation-20	High	Timing of CD-20 treatment may impact seropositivity rates; lower antibody responses were observed in patients who received treatment <6 months before vaccination compared to those who were treated over 6–12 months
Bruton tyrosine kinase inhibitor	High	Low serologic response rates but higher cellular response rates
Chimeric antigen receptor T-cell therapy	High	Rates of serologic response can remain low in patients >12 months after CAR-T therapy; studies suggest possible differences in response rates based on antibody targets (BCMA, CD-19)

Abbreviations: BCMA, B-cell maturation antigen; CAR-T, chimeric antigen receptor T-cell; CD, cluster of differentiation; ICI, immune checkpoint inhibitor; JAKi, Janus kinase inhibitor; MPN, myeloproliferative neoplasms; RBD, receptor binding domain; TKI, tyrosine kinase inhibitor.

Low impact: ≥ 80% seropositive response rate; moderate impact: 50% to 80% seropositive response rate; high impact: ≤ 50% seropositive response rate.

**Figure 1.**
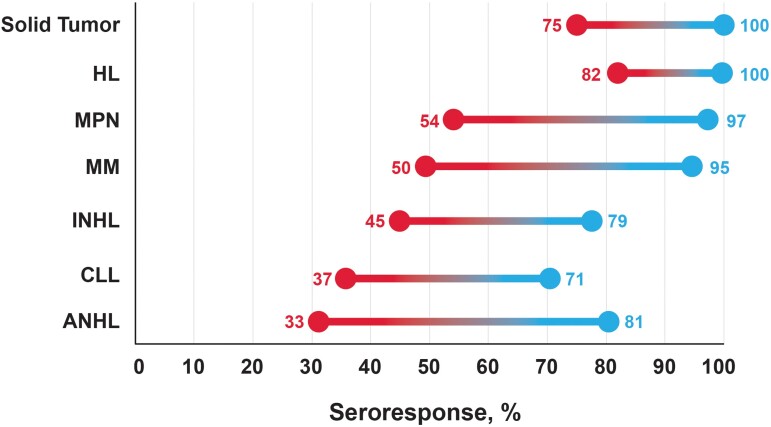
Summary of the range of pooled antibody response rates after 2 doses of COVID-19 vaccines across hematologic malignancies [38–40]. Seroresponse rates will vary depending on other factors including disease treatment status, type of therapy, and timing of vaccination. Red represents the lowest seroresponse rate observed and blue represents the highest seroresponse rate observed. ANHL, aggressive non-Hodgkin lymphoma; CLL, chronic lymphocytic leukemia; HL, Hodgkin lymphoma; INHL, indolent non-Hodgkin lymphoma; MM, multiple myeloma; MPN, myeloproliferative neoplasms.

**Figure 2.**
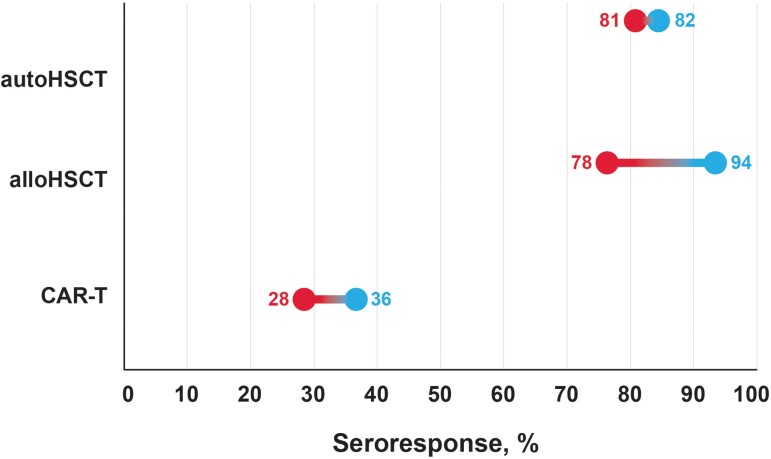
Summary of the range of pooled antibody response rates after 2 doses of COVID-19 vaccines in patients who received hematopoietic stem cell transplant (HSCT) or chimeric antigen receptor T-cell therapy (CAR-T) [16, 52–54]. Seroresponse rates will vary depending on other factors including disease treatment status, type of therapy, and timing of vaccination. Red represents the lowest seroresponse rate observed and blue represents the highest seroresponse rate observed. alloHSCT, allogeneic HSCT; autoHSCT, autologous HSCT.

Specifically, in studies of patients with solid tumors, seropositivity ranged from 75% to 100% after 2 doses of COVID-19 vaccines that received emergency use authorization from the World Health Organization, including those who received mRNA vaccination (mRNA-1273 or BNT162b2) (Figure 1 [38–40] and Tables 2 [28, 30, 38, 39, 41–47] and 3 [30, 34, 36, 38, 40, 42, 46–49, 51]) [38, 39]. Additional doses of the vaccine increased the seroresponse rates with a range of 83%–100% after dose 3% and 100% after dose 4 [38, 46, 47]. In patients with hematologic malignancies, a wide range of seropositivity rates have been reported because of populations with different underlying malignancies (Figure 1 [38–40]). Overall, rates ranged from 21% to 87% after dose 2, 14% to 96% after dose 3, and from 87% to 100% after dose 4 [30, 34, 36, 38, 40, 42, 46–51]. Rates of seropositivity after 2 doses of COVID-19 vaccine were lowest in patients with aggressive non-Hodgkin lymphoma (range, 33%–81%) and in patients with chronic lymphocytic leukemia (range, 37%–71%), whereas patients with Hodgkin lymphoma had highest response rates (range, 82%–100%). Rates of seropositivity in patients receiving HSCT therapy ranged from 78% to 94% after dose 2 and from 58% to 90% after dose 3 (Figure 2 [16, 52–54]) [16, 38, 52–55]. CAR-T recipients had the lowest rates of seropositivity, ranging from 28% to 36% after dose 2 and from 25% to 40% after dose 3 (Figure 2 [16, 52–54]) [16, 53, 55, 56].

**Table 2.** Vaccine Response in Patients With Solid Tumors

Study	Number of Doses	Study Characteristics	Vaccine(s)	Cancer Type	Seropositivity Rate^a^	nAb Response	T-Cell Response
Becerril-Gaitan et al [28]	1	Meta-analysis including 35 studies (6 studies in patients with solid tumors)	mRNA-1273, BNT162b2, ChAdOx1, Ad26.COV2.S, or CoronaVac	Solid tumors: type not specified	45%	…	78% (among 84 patients)
	2				95%	…	60% (among 634 patients)
Lee et al [41]	1	SLR (11 studies, including 827 patients with solid tumors)	mRNA-1273, BNT162b2, ChAdOx1, Ad26.COV2.S, or CoronaVac	Solid tumors: type not specified	55%	…	…
	2				90%	…	…
Corti et al [30]	2	SLR (36 studies included)	mRNA-1273, BNT162b2, ChAdOx1, Ad26.COV2.S, or CoronaVac	Solid tumors: type not specified	Overall, 85% Range^b^, 48%–100%	…	…
Martins-Branco et al [42]	2	SLR and meta-analysis including 89 records reporting data from 30 183 patients	mRNA-1273, BNT162b2, ChAdOx1, Ad26.COV2.S, or inactivated SARS-CoV-2 vaccine	Solid tumors: type not specified	Overall, 94% Range^b^, 64%–99%	…	68%
Oosting et al [39]	1	Prospective study in 503 patients with solid tumors (VOICE Study)	mRNA-1273	Bone/soft tissue Breast CNS Digestive tract Endocrine glands Female/male genital organs Head and neck Respiratory tract Skin Urinary tract	Immunotherapy, 37% Chemotherapy, 32% Chemoimmunotherapy, 33%	…	…
	2				Immunotherapy, 93% Chemotherapy, 84% Chemoimmunotherapy, 89%	…	Immunotherapy, 67% Chemotherapy, 66% Chemoimmunotherapy, 53%
Debie et al [38]	2	Prospective study in 141 patients with solid tumors or hematologic malignancies (B-VOICE Study)	BNT162b2	Solid tumors: type not specified	Targeted/hormonal therapy, 97% Chemotherapy, 75% Chemoimmunotherapy, 100% Immunotherapy, 88%	…	…
	3				Targeted/hormonal therapy, 100% Chemotherapy, 83% Chemoimmunotherapy, 100% Immunotherapy, 88%	…	…
Su et al [43]	2	Prospective study in 51 patients with solid tumors	mRNA-1273 or BNT162b2	Prostate NSCLC Colorectal Urothelial Mesothelioma Renal cell Sarcoma SCLC	53%	59%	…
	3				96%	97%	…
Fendler et al [44]	3	Prospective study in 353 patients with solid tumors or hematologic malignancies (CAPTURE study)	BNT162b2	Solid tumors: type not specified	…	nAb to delta, 67%	73%
Luangdilok et al [45]	2	Study of 131 patients with solid tumors who received homologous or heterologous dose 3	CoronaVac/ChAdOx1	Breast Colorectal Head and neck Hepatobiliary-pancreatic Esophagus/gastric Genitourinary Lung	CoronaVac/ChAdOx1, 16% ChAdOx1/ChAdOx1, 10%	…	…
	3		mRNA-1273 or BNT162b2		CoronaVac/ChAdOx1, 91% ChAdOx1/ChAdOx1, 90%	CoronaVac/ChAdOx1, 86% (omicron BA.2) ChAdOx1/ChAdOx1, 78% (omicron BA.2)	…
Debie et al [46]	3 (D 28)	Continuation of above B-VOICE study plus Tri-VOICE with 157 patients with cancer	BNT162b2	Solid tumors: type not specified	Targeted/hormonal therapy, 98% Chemotherapy, 100% Immunotherapy, 100%	…	…
	4				Targeted/hormonal therapy, 100% Chemotherapy, 100% Immunotherapy, 100%	…	…
Ehmsen et al [47]	3	Study in 395 patients with solid tumors or hematologic malignancies	mRNA-1273 or BNT162b2	Breast Urological and gynecological Skin Thoracic Gastrointestinal Other	99%	…	…
	4				100%	…	…

Additional vaccine doses can increase humoral immune response in patients with cancer who do not fully respond after 2 doses of COVID-19 mRNA vaccine and provide protection against new variants of concern (VOC). A wide range of seropositivity rates were reported, but most studies demonstrated that ≥40% of patients with cancer who did not respond to 2 vaccine doses, achieved seropositivity after an additional dose (Tables 2 [28, 30, 38, 39, 41–47] and 3 [30, 34, 36, 38, 40, 42, 46–49, 51]). Furthermore, neutralization antibody (nAb) titers increased after additional doses of COVID-19 vaccines (Tables 2 [28, 30, 38, 39, 41–47] and 3 [30, 34, 36, 38, 40, 42, 46–49, 51]). In patients with solid tumors, the percentage of patients with neutralizing responses against the omicron (B.1.1.529) variant increased from 47.8% to 88.9% after dose 3 [35]. Where measured, nAb response rates in patients with hematologic malignancies ranged from 52% to 60% after 2 doses of a COVID-19 vaccine and ranged from 62% to 100%, depending on the variant after dose 3 [40, 48, 49, 51]. Overall, additional doses (3 or 4) improved seroresponse and nAb rates, even in patients with cancer who did not respond after 2 doses.

### Cellular Immune Responses

Humoral responses after vaccination are typically reported as a measure of protection against infection; however, cellular responses can also indicate the measure of protection against infection and are associated with severity of disease. Lower T-cell responses have been found to correlate with more severe outcomes from COVID-19 [32, 57]. T-cell responses have been observed after COVID-19 vaccination despite a poor humoral immune response, and, in some patients with hematologic malignancies, T-cell response rates after vaccination may be higher than humoral response rates [48, 58–60].

Studies suggest that cellular responses were variable among patients with cancer, with T-cell responses observed in 53% to 78% of patients with solid tumors, 33% to 86% of patients with hematologic malignancies, and 42% to 72% of HSCT/CAR-T recipients (Tables 2 [28, 30, 38, 39, 41–47], 3 [30, 34, 36, 38, 40, 42, 46–49, 51], and 4 [16, 38, 52–56]). In response to COVID-19 infection, Bange et al [61] demonstrated that a greater frequency of SARS-CoV-2-specific polyfunctional (interferon-γ, interleukin 2 producing) CD8^+^ T cells correlated with survival postinfection, highlighting their potential importance following vaccination. Furthermore, T-cell responses could be crucial for protection against new viral variants [35, 62–64].

**Table 3.** Vaccine Response in Patients With Hematologic Malignancies

Study	Number of Doses	Study Characteristics	Vaccine(s)	Cancer Type	Seropositivity Rate^a^	nAb Response	T-Cell Response
Gagelmann et al [40]	1	SLR and meta-analysis including 49 studies comprising 11 086 patients	mRNA-1273, BNT162b2, ChAdOx1, or Ad26.COV2.S	Amyloidosis Lymphoma Myeloid Lymphoid Myelofibrosis CLL MM MPN NHL HL WM	Overall, 37%	…	…
	2				Overall, 64% Range^b^, 29%–87%	52%	…
Teh et al [48]	1	SLR and meta-analysis including 44 studies comprising 7064 patients	mRNA-1273, BNT162b2, Ad26.COV2.S, or ChAdOx1	Myeloma CLL Lymphoma Acute leukemia and MDS MPN and CML	Range^b^, 37%–51%	18%–63%	33%–86%
	2				Range^b^, 62%–66%	57%–60%	40%–75%
Corti et al [30]	2	SLR (36 studies included)	mRNA-1273, BNT162b2, ChAdOx1, Ad26.COV2.S, or CoronaVac	Multiple myeloma MDS MPN Lymphoma CLL	54%	…	…
Martins-Branco et al [42]	2	SLR and meta-analysis including 89 records reporting data from 30 183 patients	mRNA-1273, BNT162b2, ChAdOx1, Ad26.COV2.S, or inactivated SARS-CoV-2 vaccine	NS	Overall, 60% Range^b^, 42%–78%	…	59%
Debie et al [38]	2	Prospective study in 141 patients with solid tumors or hematologic malignancies (B-VOICE Study)	BNT162b2	NS	Rituximab, 21% Rituximab, 14%	…	…
	3					…	…
Uaprasert et al [34]	2	SLR and meta-analysis including 12 studies from 1479 patients	mRNA-1273, BNT162b2, ChAdOx1, Ad26.COV2.S, or inactivated SARS-CoV-2 vaccine	MM CLL NHL MPN HL MDS	Overall, 68% Treated, 59% Nontreated, 80%	53%	67%
	3				Overall, 41% Range^b^, 3%–83%	…	
Greenberger et al [36]	3	N = 49 patients with B-cell malignancies	mRNA-1273 or BNT162b2	CLL NHL WM MM CLL^+^ marginal zone lymphoma Epstein-Barr virus-associated lymphoproliferative disease	65%	…	…
Haggenburg et al [49]	3	Prospective cohort study in 584 patients with hematologic malignancies	mRNA-1273	Lymphoma MM CLL CML AML and high-risk MDS MPN	79%	WT, 100% Delta, 98% Omicron BA.1, 92%	…
Sherman et al [50]	3	Prospective cohort study in 94 patients with lymphoid malignancies	mRNA-1273 or BNT162b2	CLL DLBCL MCL FL MZL HL Angioimmunoblastic T-cell lymphoma CNS lymphoma PLL T-LGL	68%	…	…
Debie et al [46]	3 (D 28)	Continuation of above B-VOICE study plus Tri-VOICE with 157 patients with cancer	BNT162b2	NS	96%	…	…
	4				100%	…	…
Ehmsen et al [47]	3	Study in 395 patients with solid tumors or hematologic malignancies	mRNA-1273 or BNT162b2	CLL or SLL MM DLBCL FL MCL MZL Other	80%	…	…
	4				87%	…	…
Fendler et al [51]	3	Continuation of CAPTURE study with 80 patients with blood cancer	BNT162b2	Lymphoma Myeloma CLL Acute leukemia MDS	…	WT, 87% Delta, 72% Omicron BA.1, 62%	T-cell responses against: WT CD4^+^, 59% WT CD8^+^, 56% Omicron CD4^+^, 31% Omicron CD8^+^, 34%
	4				…	WT, 98% Delta, 78% Omicron BA.1, 79%	T-cell responses against: WT CD4^+^, 81% WT CD8^+^, 56% Omicron CD4^+^, 59% Omicron CD8^+^, 44%

Abbreviations: AML, acute myeloid leukemia; CD, cluster of differentiation; CLL, chronic lymphocytic leukemia; CML, chronic myeloid leukemia; CNS, central nervous system; DLBCL, diffuse large B-cell lymphoma; FL, follicular lymphoma; HL, Hodgkin lymphoma; MCL, mantle cell lymphoma; MDS, myelodysplastic syndrome; MM, multiple myeloma; MPN, myeloproliferative neoplasms; MZL, marginal zone lymphoma; NHL, non-Hodgkin lymphoma; nAb, neutralizing antibody; NS, not specified; NSCLC, non–small-cell lung cancer; PBMC, peripheral blood mononuclear cell; PLL, prolymphocytic leukemia; SCLC, small cell lung cancer; SFC, spot-forming cells; SLL, small lymphocytic lymphoma; SLR, systematic literature review; T-LGL, T-cell large granular lymphocytic leukemia; WM, Waldenström macroglobulinemia; WT, wild type.

Definitions for seropositivity varied across studies.

Range of seropositivity identified across studies.

**Table 4.** Vaccine Response in Patients Who Received HSCT or CAR-T

HSCT/CAR-T							
Study	Number of Doses	Study Characteristics	Vaccine(s)	Cancer Type	Seropositivity Rate^a^	nAb Response	T-Cell Response
Einarsdottir et al [52]	1	50 patients in Sweden with alloHSCT	mRNA-1273 or BNT162b2	AML ALL CLL Lymphoma MDS Myelofibrosis AA MM Thalassemia	alloHSCT, 76%	…	42%
	2				alloHSCT, 94%	…	72%
Ge et al [16]	1	Meta-analysis including 27 studies comprising 2506 patients with alloHSCT, 286 patients with autoHSCT, and 107 patients with CAR-T	mRNA-1273, BNT162b2, Ad26.COV2.S, or ChAdOx1	Multiple	Overall, 62% Range, 17%–89% autoHSCT, 87% alloHSCT, 59% CAR-T, 20%	…	…
	2				Overall, 75% Range, 7%–97% autoHSCT, 82% alloHSCT, 79% CAR-T, 28%	…	…
	3				Overall, 69% Range, 7%–97% autoHSCT, 63% alloHSCT, 82% CAR-T, 25%	…	…
Wu et al [53]	1	Meta-analysis including 44 studies comprising 3495 patients with alloHSCT, 1182 patients with autoHSCT, and 80 patients with either alloHSCT or autoHSCT, and 12 studies with 174 patients with CAR-T	mRNA-1273, BNT162b2, ChAdOx1, Ad26.COV2.S, or CoronaVac	NS	autoHSCT/alloHSCT, 41%	…	…
	2				autoHSCT/alloHSCT, 81% CAR-T, 36%	…	…
	3				autoHSCT/alloHSCT, 79%	…	…
Shem-Tov et al [54]	2	152 patients in Israel with alloHSCT	BNT162b2	AML MDS MPN ALL NHL HL CLL AA	alloHSCT, 78%	HSCT GMT, 116 Healthy individuals GMT, 428	…
Debie et al [38]	2	Prospective study in 141 patients with solid tumors or hematologic malignancies (B-VOICE Study)	BNT162b2	NS	HSCT, 90%	…	…
	3				HSCT, 90%	…	…
Abid et al [55]	3	A total of 75 patients (alloHSCT, n = 30; autoHSCT, n = 26; CAR-T, n = 10) were included	mRNA-1273 or BNT162b2	NS	autoHSCT, 63% alloHSCT, 58% CAR-T, 40%	…	…
Sesques [56]	3 or 4	43 CAR-T patients in France	mRNA-1273 or BNT162b2	DLBCL FL Other NHL	CAR-T, 18%	…	42%

Abbreviations: AA, aplastic anemia; ALL, acute lymphoblastic leukemia; alloHSCT, allogeneic hematopoietic stem cell transplant; AML, acute myeloid leukemia; autoHSCT, autogeneic hematopoietic stem cell transplant; CAR-T, chimeric antigen receptor T-cell therapy; CLL, chronic lymphocytic leukemia; DLBCL, diffuse large B-cell lymphoma; FL, follicular lymphoma; GMT, geometric mean titer; HL, Hodgkin lymphoma; HSCT, hematopoietic stem cell transplant; MDS, myelodysplastic syndrome; MM, multiple myeloma; MPN, myeloproliferative neoplasms; nAb, neutralizing antibody; NHL, non-Hodgkin lymphoma; NS, not specified.

Definitions for seropositivity varied across studies.

### Durability of the Immune Response

Humoral responses are generally less durable in patients with cancer than in healthy populations [65]. The reduced durability of COVID-19 vaccine-induced immunity may be attributed to a lack of memory cell formation in these populations, or possibly from ongoing B-cell depletion from certain therapies in B-cell hematologic malignancies [35, 65]. Binding and nAb waning are more often observed among patients with hematologic malignancies than those with solid tumors [37, 65]. A study in patients with cancer undergoing systemic treatment or HSCT observed that the anti-RBD response peaked 4 weeks after dose 2 of an mRNA vaccine and was sustained at 6 months after dose 2, although peak antibody levels in these patients were well below antibody concentrations observed in healthy participants [65]. The durability of immune responses after additional doses of COVID-19 vaccines is still under investigation and will be an important factor in timing and administration of boosters.

### Approaches to Enhancing the Immune Response to SARS-CoV-2 Vaccination Beyond Additional Doses

Increasing the number of vaccine doses administered to patients with cancer has demonstrated beneficial effects on both the humoral and cellular response; however, from a practical standpoint, it may not always be feasible to frequently administer doses in these patients, especially with ongoing active treatment. Additional strategies are needed to bolster the immune response in this population without solely relying on administering more doses. Alternative strategies that have been explored to enhance the immune response after COVID-19 vaccination include extending the interval between doses and using heterologous vaccination (administration of different vaccines, including those from different vaccine platforms). Note that a delayed interval dosing has been evaluated in a few studies of healthcare workers but not in cancer populations. Studies in healthcare workers found that extending the standard 3- to 6-week interval between the first and second doses of BNT162b2 to 6 to 14 weeks or 8 to 16 weeks significantly increased anti-RBD antibody titers, improved nAb responses to ancestral, alpha, beta, and delta strains, and enriched the CD4^+^ and CD8^+^ T-cell response [66, 67].

Use of a heterologous vaccine regimen can enhance humoral and cellular response in patients with cancer as well as healthy individuals. In particular, in patients with solid tumors who received a heterologous regimen of CoronaVac/ChAdOx1 vaccines for doses 1 and 2 followed by a third dose with an mRNA vaccine, seroresponse increased from 16% after dose 2 to 91% after dose 3 with significant increases in anti-RBD antibodies observed [45]. A study in patients with hematologic malignancies investigated the serologic response to a third dose of Ad26.COV2.S after 2 doses of BNT162b2 and observed a seroresponse in 31% of patients [68]. In immunocompetent individuals, a heterologous vaccine regimen increased nAb titers by a factor of 4 to 73 as well as increased spike-specific CD4^+^ and CD8^+^ T-cell responses, providing a robust and durable immune response [69]. These strategies offer several options to help enhance the immune response in patients with cancer and need to be further evaluated.

Careful consideration should be used when determining the number of doses, the interval between doses, and the type of vaccine administered regarding both the primary series (doses 1 and 2) as well as any subsequent additional doses (dose 3 and beyond). The development and availability of variant-updated booster vaccines can potentially expand the protection against SARS-CoV-2 VOC. The bivalent vaccines combining ancestral and an omicron variant strain have yet to be robustly evaluated in patients with cancer.

## REAL-WORLD EFFECTIVENESS

Although patients with cancer generally develop a less robust immune response after vaccination against COVID-19 compared to individuals without cancer [35], the risk of SARS-CoV-2 infection and severe COVID-19 among patients with cancer is lower after completion of 2 doses of an mRNA vaccine, with or without additional doses, than in unvaccinated patients with cancer [23, 70]. Effectiveness of a third mRNA vaccine dose against breakthrough infections, symptomatic infections, COVID-19–related hospitalization, and mortality in patients with cancer was 59.1%, 62.8%, 80.5%, and 94.5%, respectively [71]. In a large, real-world study, the vaccine effectiveness (VE) of mRNA vaccines against COVID-19 hospitalization in patients with cancer (before omicron variants became predominant) was estimated to be 75% [35]. Another study in the United Kingdom reported the VE of available COVID-19 vaccines against breakthrough infections in patients with cancer was similar to a control population shortly after dose 2 (65.5% vs 69.8%); however, VE at 3–6 months after dose 2 was lower in the cancer cohort than the control population (47.0% vs 61.4%) [72].

Consistent with observations in the general population, VE against VOCs have also decreased progressively in patients with cancer, although the reduction has been most prominent in patients with hematologic malignancies [35]. A study demonstrated 56% of patients with hematologic malignancies had neutralizing activity against the ancestral Wuhan strain but only 31% of patients had detectable antibody concentrations against the delta VOC after 2 vaccine doses [73]. Although mRNA COVID-19 vaccines offer some protection against breakthrough infections, the probability of these infections increases over time due to waning immunity. Breakthrough infections in patients with cancer often have a more severe course and a higher risk of mortality compared with a healthy population [35]. With the ever-evolving landscape of SARS-CoV-2 VOC and the impact of waning immunity, it remains vital to continue assessing the VE of COVID-19 vaccines, including the variant-updated bivalent vaccines, in patients with cancer.

## SAFETY OF COVID-19 VACCINES IN PATIENTS WITH CANCER

Although patients with cancer were not included as the majority targeted population in the original registry trials of COVID-19 vaccines, the safety profile of these vaccines in immunocompromised populations has since been established [15]. At this time, there is no evidence to suggest that COVID-19 vaccines have a different safety profile in patients with cancer than in the general population, and patients receiving cancer therapies do not have an increased risk of immune-related adverse events (AEs) or graft-versus-host disease (hematologic malignancies) after administration of a COVID-19 vaccine [15, 35, 52]. Furthermore, no differences in vaccine-related AEs have been observed between hematologic or solid malignancies or between patients undergoing cancer treatment and those who were treatment naive [15]. The most common local and systemic side effects related to vaccination were pain at injection site, myalgia, and fatigue, which were mostly mild to moderate in severity. In patients receiving HSCT or CAR-T, common local reactions related to vaccination included pain and swelling and redness at the injection site; common systemic reactions included fever, chills, fatigue, myalgias, and arthralgias [16]. These events were generally mild and resolved within days. Overall, the safety of COVID-19 vaccines in patients with cancer is comparable to that of the general population and the benefits of COVID-19 vaccination greatly outweigh the risks for patients with cancer [35].

## CONCLUSIONS

As COVID-19 continues to evolve, it is important to continually evaluate the immune response and real-world effectiveness of vaccination among immunocompromised individuals. Patients with cancer typically develop a less robust immune response after COVID-19 vaccination than individuals without cancer, resulting in higher risk for SARS-CoV-2 infection and severe COVID-19 [35]. In general, lower immune responses are observed in patients with hematologic malignancies compared with patients with solid tumors [15]. However, cellular responses, which are associated with protection against severe disease, are higher than humoral response in some patients with hematologic malignancies and B-cell depletion, which highlights the ongoing importance of vaccination in these groups [52, 53].

In most severely immunocompromised patients, a 3-dose primary series of an mRNA vaccine is recommended followed by additional booster doses. Recommendations for bivalent vaccines vary by country and region. Administration of additional doses may induce stronger humoral and/or cellular responses in these vulnerable populations with preliminary evidence of translation to real-world effectiveness [71]. Although both humoral and cellular responses tend to increase with additional doses of vaccine, it is important to note that cellular responses can still occur in the absence of a humoral response and could offer protection against severe COVID-19 [59–61].

Adverse event rates related to vaccination are generally low in patients with cancer, demonstrating that these individuals can be safely vaccinated during active therapy [15]. Furthermore, patients with solid tumors can generally achieve humoral and cellular response rates that are similar to those responses in immunocompetent individuals [15, 35]. However, in some patient groups, low or nonexistent humoral response rates are observed, and additional preventative measures may be required [16, 38, 59, 74].

Future studies should focus on identifying correlates of protection for vaccinated individuals, especially among those with poor humoral responses. In addition, improved reporting of cellular responses in patients with cancer is needed to better understand the immune responses observed in this population. It would also be important to examine whether the immune response recovers during remission and to what extent. Finally, more data are needed on heterologous vaccination schedules and varied dosing intervals in immunocompromised populations. This review was limited in focus on the immunologic and clinical responses to COVID-19 vaccination in patients with solid tumors and hematologic malignancies. Additional studies on the immune response after variant-updated bivalent vaccines and vaccination in combination with COVID-19 therapeutics or other types of pre-exposure prophylaxis are needed in patients with cancer.

## Notes

***Acknowledgments***. Medical writing and editorial assistance were provided by Lindsey Kirkland, PhD, of MEDiSTRAVA in accordance with Good Publication Practice (GPP 2022) guidelines, funded by Moderna, Inc., and under the direction of the authors.

***Supplement sponsorship.*** This article appears as part of the supplement “COVID-19 Vaccination in the Immunocompromised Population,” sponsored by Moderna, Inc.
